# The Mental Health Consequences of Work-Life and Life-Work Conflicts for STEM Postdoctoral Trainees

**DOI:** 10.3389/fpsyg.2021.750490

**Published:** 2021-11-16

**Authors:** Richard N. Pitt, Yasemin Taskin Alp, Imani A. Shell

**Affiliations:** Department of Sociology, University of California, San Diego, San Diego, CA, United States

**Keywords:** work-life imbalances, postdoctoral trainees, academic workers, mental health, anxiety, attrition, persistence

## Abstract

Research has shown that work-life conflicts exist among all kinds of workers, including academics, and these conflicts are a key contributor to workers’ reports of poor well-being. Very little research has been done on work-life conflict among post-baccalaureate PhD trainees (e.g., graduate students and postdoctoral trainees) who reside in an important liminal stage in the professoriate pipeline. In this study, we examine the degree to which postdocs believe they suffer from conflicts between their work responsibilities and their home responsibility and the relationship between those conflicts and postdoc’s mental health. We argue that, like other workers, postdocs suffer (in numerical terms and its relationship to health) more from the work-to-life imbalances than from life-to-work imbalances; life matters more than work, ultimately. Our results, based on a survey of 215 STEM postdoctoral trainees, reveal that a majority of postdocs say they have work-life conflicts and these work-life conflicts are associated with negative mental health outcomes. We discuss the potential impact of these findings on attempts to broaden participation in STEM careers and diversify the professoriate.

## Introduction

Work-life conflict occurs when work responsibilities and life (usually household) responsibilities interfere with each other (Greenhaus and Beutell, 1985; Frone, 2000; Amstad et al., 2011).^1^ For example, workers have less time to spare for work or life (Netemeyer et al., 1996; Carlson and Perrewe, 1999; Edwards and Rothbard, 2000; Buonocore and Russo, 2013), stressors in one sector often impact performance in the other (Byron, 2005), and the different roles in the two arenas may not be easy to separate from one another (Olson-Buchanan and Boswell, 2006). These conflicts are key contributors to reports of poor well-being among workers (Burke, 1988; Frone, 2000; Grant-Vallone and Donaldson, 2001; Grzywacz and Bass, 2003; Denson et al., 2018) as well as low productivity and turnover in jobs (Poulose and Sudarsan, 2014; Badri, 2019).

Moreover, studies detailing the impact of work-life conflicts on well-being among all kinds of workers have led researchers to take an interest in the work habits of college and university faculty given the nature of their jobs and research suggesting faculty work anywhere from 50 to 60h each week (Jacobs and Winslow, 2004; Misra et al., 2012). These investigations have revealed that, despite the autonomy and flexibility of work that characterizes this occupation, faculty too (particularly those in STEM fields) report suffering from work-life imbalances. Ironically, those two amenities of academic work – autonomy and flexibility – lead to overworking and overlaps between work roles/responsibilities and those responsibilities faculty may have in their households (Williams, 2000; Fox et al., 2011; Culpepper et al., 2020). Research on the work-life balance experienced by faculty has revealed that work-life balance is crucial to their well-being as well (Damaske et al., 2014; Ren and Caudle, 2016). This research has proven valuable and enlightening, igniting important conversations on university campuses about ways to ameliorate what is often seen as an intractable problem in academic environments.

Like the research on faculty, most research on work-life conflict has focused on employed laborers, that is, workers in the part-time or full-time paid workforce. Virtually, no attention has been paid to work-life conflict of trainees in the pipeline to some of these positions, especially when those trainees have considerable time commitments as working apprentices.^2^ Specific to this paper, few studies have tested the relationship between work-life balance and well-being among postdoctoral trainees (going forward, “postdocs”) who sit in the liminal stage between graduate school and being faculty (Moors et al., 2014; Ysseldyk et al., 2019). It is important to understand the lives of postdocs because their experiences, like those of graduate students, are often critical to the decision to pursue academic careers. If postdocs are experiencing work-life conflicts and the concomitant impacts of those conflicts on their mental health, we might expect them to be less productive in their postdoc appointments (and thereby, less competitive for jobs) and/or more likely to want to leave the academic pipeline, another version of professional turnover. It is, therefore, important to know more about the experience of work-life conflict among postdoctoral trainees.

We will fill this gap in the literature by scrutinizing the relationship between work-life conflict and mental health among the postdocs by using a survey of 215 STEM postdoctoral trainees. We look into both work-to-life conflict (i.e., the demands of work interfere with home/family life) and life-to-work conflict (i.e., the demands of family or spouse/partner interfere with job-related activities). We seek to answer two related questions: a) whether work-life conflicts exist (in both directions) for postdocs and b) whether those conflicts predict higher levels of anxiety while controlling for the postdocs’ background, health status, and their experience in their postdoc appointment.

In the following pages, we first outline the literature on work-to-life conflict and its impact on mental health. Next, we describe our data and methods and present correlations between our variables. We then report our regression results that shows that work-life conflicts significantly impact postdocs’ mental health. Finally, we discuss our research’s findings and implications for postdoctoral trainees’ transition to academic jobs.

## Background

### Work-Life Conflicts and Imbalances

Both family and work have gone through significant transformations in the past two centuries, along with shifts in the demographics of those making up the labor force. These resulted in increases in the time people spend working and “changes in the pace and intensity of work” (Kossek et al., 1999; Helmle et al., 2014). Furthermore, the number of households with dual incomes increased, more women are in the workforce, and the proportion of older people in the population is higher (Hammer et al., 2005). In addition to these major social changes, researchers have been interested in studying how workers manage their work and life because it can provide valuable information concerning workers’ mental health and life satisfaction, which ultimately affects their productivity at work and job satisfaction (Poulose and Sudarsan, 2014).

Work-life balance refers to the harmony one achieves when their work does not interfere with the activities and roles they have outside of work. Work indicates a job someone does to pay for their livelihood. The non-work (i.e., “life”)-related activities they do could range from taking care of their children and spending time with their partner to enjoying leisure and avocational activities. Work-life balance consists of a person’s perception of the amount of time that is available to them for both their work and life outside of work (Gröpel and Kuhl, 2009). When there is an imbalance caused by one sector, it is likely to negatively affect the other sector (Poulose and Sudarsan, 2014). A work-life imbalance – or conflict – means that one’s work duties inhibit their ability to fulfill their life duties and/or one’s life duties affect their work duties (Greenhaus and Beutell, 1985; Frone, 2000; Amstad et al., 2011).

Greenhaus and Beutell (1985) outline three reasons why people have work-life imbalances. The first reason is the time-based conflict which states that one’s time commitment to and demands of one role leaves little room for completing the other role (Netemeyer et al., 1996; Carlson and Perrewe, 1999; Edwards and Rothbard, 2000; Buonocore and Russo, 2013). Three examples of this type of conflict would involve an excessive amount of time spent working a week, having a work schedule that is not flexible, and “role overload” (Keith and Schafer, 1980; Pleck et al., 1980; Burke, 1988). The second reason is the strain-based conflict which states that the resulting strain (i.e., tension or anxiety) from one role complicates the ability to fully perform another role (Netemeyer et al., 1996; Edwards and Rothbard, 2000; Byron, 2005; Buonocore and Russo, 2013). Lastly, the third reason is the behavior-based conflict which claims that the specific behaviors required of one role are not compatible with another role (Edwards and Rothbard, 2000; Buonocore and Russo, 2013).

Researchers study work-life balance because it affects workers’ overall well-being. Work-life conflicts a strong positive relationship with psychiatric disorders, such as anxiety, mood, and substance addictions (Burke, 1988; Frone, 2000; Grant-Vallone and Donaldson, 2001; Grzywacz and Bass, 2003; Bellavia, 2005; Amstad et al., 2011). For instance, Frone’s (2000) study of the work-life conflict of employees demonstrated that those with this type of conflict had an increase of 1.99 to 29.66 times the likelihood to have a mental illness than those without this type of conflict. This has implications for the person’s ability to fully show up for the various roles in their life, let al. one sustain a healthy lifestyle. Conversely, more work-life balance is associated with good mental health and lower rates of turnover (Badri, 2019). By studying the nuances of the work-life balance, research has uncovered how workers’ roles on an individual and organizational level influence their mental health (Hammer et al., 2005; Badri, 2019).

### Faculty and Work-Life Conflict

Research shows that college and university faculty, particularly STEM faculty, tend to work longer hours than people in other professions, leaving them with less time to spend on their non-work life (Jacobs, 2004). In line with this, people often go into the faculty pipeline with the understanding that at some point, they may be confronted with high expectations on the number of hours they need to work. The work faculty do is closely linked to their identity, which has implications for the amount of time they dedicate to their work (Fox et al., 2011; Lester, 2013). With an academic culture that rewards faculty who work overtime and constantly perform at high levels, there is no surprise that many find themselves stretched for time to do things outside of work (Fox et al., 2011). However, although academia may require long work hours, it is also one of the most flexible fields to work in (Damaske et al., 2014; Fontinha et al., 2019). As such, faculty should still be able to adjust their schedules to fit the needs of their non-work life. These characteristics make the study of the work-life balance of academics extremely valuable for researchers in higher education. Like other workers, when faculty have work-life imbalance, they experience various psychological and emotional illnesses. Faculty with work-life conflicts are more likely to report mental health problems, low satisfaction with their work, and a higher propensity for burnout and decisions to leave their positions (Aazami et al., 2015; Kazley et al., 2016; Denson et al., 2018; Badri, 2019). Given the correlation between work-life balance and mental health among faculty, it is possible that others in academia, especially those in the pipeline to be faculty, may have similar difficulties managing their work/life conflicts and suffer negative mental health outcomes as well.

### Postdoctoral Trainees, Work-Life Conflict, and Mental Health

Most, if not all, of the literature on work-life imbalances and health focuses on full-time workers, including faculty. Very little attention has been paid to the potential for work-life conflict encountered by trainees for faculty careers (e.g., graduate students and postdoctoral associates) who also work part- or full-time schedules as part of their training regimen. While some research (Schwartz et al., 1990; Dorsey et al., 2003; Tambyraja et al., 2008) has documented the problems, medical residents have achieved a balance between work and family/personal time – problems leading to declines in interest in some medical specialties (e.g., surgery) – virtually, no research has examined work-life conflicts among STEM postdocs, who likely experience similar difficulties. The little research that has been done suggests that postdocs, like medical residents and faculty, find it difficult to attain work-life balance, and this likely leads to negative mental health outcomes (Ysseldyk et al., 2019).

As taking on a postdoctoral appointment is becoming almost normative in STEM disciplines, particularly in the biological and biomedical sciences, understanding the behaviors, motivations, and experiences in this liminal – and for many, pivotal – stage in the STEM professoriate pipeline is important for building a more complete picture of what causes attrition from and persistence in that pipeline, particularly for future faculty of color and women.

As the stage in a potential faculty member’s training that most closely approximates what it might be like to *be* faculty, we suspect the difficulty faculty have maintaining balance between work demands and the demands/desires associated with their non-work life will be found among postdoctoral trainees as well. The association between these work-life conflicts and mental health is well-documented. Therefore, we make the following hypotheses:

*H1*: A majority of STEM postdoctoral associates and fellows experience conflicts between their work demands and the home/life responsibilities. This takes three forms:

*H1a*: A majority of postdocs – regardless of family status – will report that the demands of their job interfere with their home and/or family life (work-to-life conflict).

*H1b*: Among postdocs with families (i.e., spouse/partner, children), a majority will report that their home life interferes with their job-related activities and responsibilities (life-to-work conflict).

*H1c*: Among postdocs without families, few will report that their home life interferes with their job-related activities and responsibilities (life-to work conflict).

*H2*: Work-life conflicts are a predictor of poor mental health. This relationship exists for both work-to-life conflicts and life-to-work conflicts.

*H2a*: The greater one’s experience of work-to-life conflicts, the higher the levels of mental health disorder they will report.

*H2b*: The greater one’s experience of life-to-work conflicts, the higher the levels of mental health disorder they will report.

## Data and Methods

We used a web-based survey as the principal tool to gather information from 215 STEM postdoctoral appointees. In 2017, staff members in the Offices of Postdoctoral Affairs (OPA) at 30 research-intensive doctoral universities forwarded our invitation to participate in the research to their cohort of postdoctoral trainees.^3^ The invitation described the parameters for involvement in the research, specifically, that potential respondents be US citizens or permanent residents in the first, second, or third year of their first postdoctoral appointment in one of five broad STEM categories: agriculture and conservation resources, biological and biomedical sciences, STEM education, engineering and computer science, or the physical sciences and math.^4^ First-time postdocs were chosen because we were interested in the pathway from receipt of doctorate through the first postdoc position to faculty, other postdoc positions, or non-academic jobs. The OPA staff were informed that we were particularly interested in understanding the experiences of women; as a result, this population was oversampled.

While an accurate accounting of how many potential respondents were exposed to the recruitment materials was unavailable to us, more than 750 postdocs responded positively to the invitation. Most of those potential respondents were ineligible to participate because they did not meet the base requirements for inclusion in the study. Ultimately, we ended with a sample of 215 postdoctoral trainees. Of these respondents, 65% are women. We weighted our analyses to account for the oversampling that created this conflict. We used the proportion of STEM postdoctoral recipients (35%; National Center for Science and Engineering Statistics, 2017a) who are women as a target population for this weighting. The racial balance – 77% White, 23% non-White – more closely approximates the percentages of White/non-White US citizens and permanent residents with STEM doctorates in the disciplines we analyze (National Center for Science and Engineering Statistics, 2017b).^5^ More than half (51%) of our respondents were in their of the postdoc. Representation among the disciplines was as follows: agriculture (6.5%), biological and biomedical sciences (56.3%), STEM education (3.3%), engineering (14.4%), and physical sciences (19.5%); these percentages differ from the national postdoc population by less than 10% (National Center for Science and Engineering Statistics, 2017a).

In addition to the survey data on which this analysis is based, we conducted interviews (*n*=75) with survey respondents about their first-year experiences of their postdoctoral appointments. The majority (77%) of these interviews involved some discussion of either the work-life conflicts postdocs were experiencing themselves or their assumptions about work-life conflicts awaiting them if they pursue careers in the academy. These responses were most commonly a response to the question, “What is your current experience like in laboratories and with faculty advisors.” While not used in the primary analysis reported here, selected quotes from these interviews will be used in the discussion to contextualize recommendations to ameliorate these conflicts.

### Key Independent Variables: Work-Life/Life-Work Conflicts

We were interested in examining the relationship between mental health outcomes and conflicts between our respondents’ work responsibilities and their home/family life. Therefore, we used two series of four questions developed by Netemeyer et al. (1996) that measure conflicts between one’s work and home life responsibilities. These scales reliably capture the various ways work might impact or be impacted by ones’ responsibilities at home, be they family-related (e.g., partners, children, and elder care.) or not.

The first independent variable is work-to-life conflict. On a 4-point scale ranging from 1 (strongly disagree) to 4 (strongly agree), respondents indicated the extent of their agreement with the following four statements: “The demands of my job interfere with my home and family life,” “The amount of time my job takes up makes it difficult to fulfill my family responsibilities,” “Things I want to do at home do not get done because of the demands my job puts on me,” and “Due to job-related activities, I have to make changes to my plans for family activities.” These items were combined in a scale ranging from 4 (complete strong disagreement) to 16 (complete strong agreement). This scale has a mean value of 10.24 and is treated as a single factor: work-life conflict (*α*=0.86).

The second independent variable is life-to-work conflict. On a 4-point scale ranging from 1 (strongly disagree) to 4 (strongly agree), respondents indicated the extent of their agreement with the following four statements: “The demands of my family or spouse/partner interfere with my job-related activities,” “I have to put off doing things at my job because of demands on my time at home,” “Things I want to do at my job do not get done because of the demands of my family or spouse/partner,” and “My home life interferes with my responsibilities at my job such as getting to work on time, accomplishing daily tasks, and working overtime.” These items were also combined in a scale ranging from 4 (complete strong disagreement) to 16 (complete strong agreement). This scale has a mean value of 8.04 and is treated as a single factor: life-work conflict (*α*=0.87).

### Key Dependent Variables: Generalized Anxiety Disorder

We use generalized anxiety disorder as our measure of mental health disorder. Generalized anxiety disorder is measured using 7-item Generalized Anxiety Disorder scale (GAD-7; Spitzer et al., 2006). The GAD-7 only estimates symptoms of anxiety. On a 7-point scale ranging from 0days to 7days, trainees indicated the number of days in the past week that they felt such experiences as “worried too much about little things” and “felt so restless that it is hard to sit still.” These items were combined in a scale ranging from 11 to 40 (*x̄*=21.75) and treated as a single factor: anxiety (*α*=0.82). If we divide the scale by the number of statements (7) and identify which respondents experience the set of seven symptoms more than 3days a week, we determine that 34% of our respondents experience the amount of anxiety deemed problematic.

### Demographic Controls and Other Likely Covariates

We control for seventeen factors that may covary with these mental health outcomes. These factors are added into the models in three sections: health covariates, common demographic covariates, and variables related to respondents’ STEM training and experience.

Self-reported overall health was measured using one-item: “Thinking back over the past month, how would you say your general health has been?” Trainees could choose poor, fair, good, very good, and excellent. This seemingly simple self-rating of general health has been shown to be more reliable than even physicians’ ratings of health (Idler and Benyamini, 1997). We recoded this variable to a dummy variable such that “1″ represents “very good” and “excellent” health (*x̄*=0.52). We also include two other personality variables that are associated with mental health: dispositional optimism (i.e., an inclination to have favorable expectations for one’s future regardless of the odds) and mastery (i.e., sense of control over one’s life). We use Scheir and Carver’s (1985) 12-item Life Orientation Test for dispositional optimism: Respondents indicate degree of agreement with statements, such as “I’m a believer in the idea that every cloud has a silver lining” (range=10–32, *x̄*=21.81, *α*=0.83). We use the 7-item Pearlin and Schooler (1978) Mastery scale for mastery: Respondents indicate degree of agreement with statements, such as “I have little control over the things that happen to me.” (range=11–28, *x̄*=20.60, *α*=0.80).

The second group of possible covariates includes demographic characteristics commonly associated with academic/occupational identity and/or mental health among postdoc populations:

gender (female=1; *x̄*=0.35), race (non-White=1; *x̄*=0.21), age (continuous variable; *x̄*=31.82), high levels of educational debt (debt over $40k=1; *x̄*=0.13), and high household income (income over $100k=1; *x̄*=0.30). Two demographic covariates – relationship status (coupled=1; *x̄*=0.75)^6^ and parenthood (parent=1; *x̄*=0.18)—are analyzed separately to answer hypotheses 1a–1c. They are also used in the regressions as controls.

We then control for six variables reflecting experiences gained in the pursuit of their training in science. Our respondents represent a range of one to three years in their postdoc; we control for year one (*x̄*=0.54). Using a set of questions commonly used to determine positive appraisals (e.g., “my colleagues view me as a scientist,” “my supervisor(s) view me as a scientist”), we created a scale (range=10–36, *x̄*=25.78, *α*=0.90) indicating the degree to which respondents agree various communities recognize them as a scientist; we refer to this as “science appraisals.” We also include a science efficacy scale (range=26–48, *x̄*=41.31, *α*=0.84) from a set of questions asking respondents to indicate their level of confidence in their ability to perform twelve science tasks (e.g., use technical instruments and techniques and report research results in a written paper). Perceived unfair treatment was measured using the Everyday Discrimination Scale (EDS; Williams and Mohammed, 2009) with a 7-item scale (range=7–32, *x̄*=13.07, *α*=0.83) where respondents indicated how often (never to almost every day) they had experienced some form of treatment they perceived as unfair or unwarranted in their workplace (e.g., “You have been treated with less respect than expected at work,” “Individuals at your institution acted as if they think you are not smart”).^7^ Success in publishing and presenting research should support positive mental health outcomes for academic trainees. If respondents have published solo authored papers, presented solo authored papers, or have won awards, we coded them “1”; “0” if they do not report these successes (*x̄*=0.62). The models also included a dummy variable where “1” represents if they have a faculty mentor (*x̄*=0.79).

### Analytical Strategy

We used ordinary least squares regression modeling in order to determine the relationship between our independent variables (work-life/life-work conflict) and generalized anxiety.

## Results

If we divide both work-life/life-work conflict scales by the number of statements (4) and identify which respondents have an average response greater than 2.5 (agree and strongly agree), we determine that nearly 62% of our respondents experience work-life conflict. About 15 percent of our respondents experience a high (1 standard deviation above the mean) level of work-life conflict. Surprisingly, there is no significant difference between singles and coupled respondents in the degree to which they report work-life conflicts. There are also no differences between respondents who have children and those who do not. In the aggregate, far fewer postdocs report experience life-work conflict; only 25% do. Sixteen percent of our respondents experience a high (1 standard deviation above the mean) level of life-work conflict. There are statistically significant differences between single respondents (7%) and coupled respondents (25%) in regard to their reports of life-work conflicts. A much more substantial difference exists between non-parents (12%) and non-parents (58%); 50 % of parents report *high* levels of life-work conflict. These findings support the three H1 hypotheses. The bivariate correlations between coupled/parent status and the work-life/life-work conflict scales (i.e., the entire range of 4–16) used in the regression analyses are described below.

### Bivariate Correlations

Table 1 presents bivariate correlations between the independent (work-life conflict and life-work conflict), dependent (generalized anxiety disorder), and selected control variables. We include these correlations both as a presaging of what we are likely to discover about the relationships between the independent/control variables and anxiety and as an opportunity to examine any relationships that might exist between those variables and each other. Certainly, we expect to see that there is a positive correlation between the two kinds of conflict, but there is some value in determining if some postdoc characteristics (e.g., their gender, race, or parental status) are predictive of the degree of work-life and life-work conflict they might experience.

**Table 1:** Bivariate correlations between dependent (anxiety), independent (work-life conflict and life-work conflict), and selected control variables.

	Work-life conflict	Life-work conflict	Anxiety
Work-life conflict		0.430^***^	0.393^***^
Life-work conflict	0.430^***^		0.238^***^
General health	−0.201^**^	−0.186^**^	−0.368^***^
Dispositional optimism	−0.136^*^	−0.129	−0.428^***^
Personal locus of control	−0.113	−0.081	−0.502^***^
Female	0.101	−0.011	0.088
Non-white	0.013	−0.084	−0.015
Coupled	−0.024	0.221^**^	−0.109
Parent	0.049	0.431^***^	−0.069
Educational debt is high	0.142^*^	−0.059	0.090
Science community	−0.106	−0.105	−0.266^***^
Science efficacy	0.044	−0.090	−0.141^*^
Has a faculty mentor	−0.163^*^	−0.075	−0.166^*^
Experienced job successes	−0.032	−0.068	−0.060
Experienced unfair treatment	0.352^***^	0.222^***^	0.395^***^

N=215. * *p<0.05;*

** *p<0.01;*

*** *p<0.001.*

Looking across the rows, the two kinds of conflict are associated with each other and, as predicted, are positively associated with anxiety: The higher one’s work-life and life-work conflicts, the higher they are on our measure of generalized anxiety. The other three positive health variables (general health, optimism, and mastery) are all negatively correlated with anxiety. General health is negatively correlated with both kinds of work/life conflicts: The greater the conflict, the lower one’s general health. This relationship exists with dispositional optimism, but only with work-vs.-life conflicts.

Of the demographic characteristics, only two are associated with mental health: Being coupled is negatively associated with anxiety, while having high levels of educational debt is positively associated with anxiety. As described previously, family status is unrelated to work-vs.-life conflicts, but positively associated with life-vs.-work conflicts. Parenthood is positively associated with life-vs.-work conflicts, but not anxiety.

Science community appraisals and science efficacy (both, presumably, and positive holdings) are almost consistently^8^ associated with lower reports of mental health disorder. Having a faculty mentor also seems to be protective against all four mental health disorder; it is negatively associated with work-life conflicts. Experiences of success (e.g., publishing sole authored papers) are negatively associated with anxiety as well.

Experiences of unfair treatment – which may not always be reflective of an experience of racial/gender discrimination – are positively associated with anxiety and, interestingly, also positively associated with both kinds of work/life conflicts. While our cross-sectional analysis cannot prove a causal ordering here, we suspect this latter association is likely a sign of high-effort coping, a response sometimes referred to as John Henryism when describing the response in African-Americans (James, 1994). James and colleagues developed the John Henry^9^ concept in an effort to describe an “individual’s self-perception that he can meet the demands of his environment through hard work and determination” (James et al., 1983, p. 263). In the face of blocked opportunities – represented by the Everyday Discrimination scale – our respondents overextend themselves at work in ways that place additional strain on their ability to fulfill their household responsibilities; this creates work-to-family conflict. Likewise, their household responsibilities hinder these attempts to overextend themselves, creating family-to-work conflict.

### Multivariate Regressions

In this section, we turn to multivariate analyses of the relationships between work-life conflicts and anxiety. In these analyses, we examine the covariates alone and then add work-to-life conflict and life-to-work conflict in separate analyses.

#### Control Variables

In Table 2, Column I, we provide a reduced model that includes only the controls. Column I reveals obvious associations between some control variables and some surprising lack of associations between others. This model appears to be fairly comprehensive, with explanatory power of 39.4 percent for anxiety symptoms.

**Table 2:** Multivariate regression testing the predictive relationship of work-life/life-work conflict on the mental health of stem postdoctoral trainees. Standardized betas reported.

	Covariates only	Work-life conflict	Life-work conflict	Both conflicts
	I	II	III	IV
Work-life conflict		0.237^***^		0.215^***^
Life-work conflict			0.156^*^	0.554
Health covariates				
General health	−0.235^***^	−0.202^***^	−0.211^***^	−0.197^***^
Dispositional optimism	−0.130	−0.114	−0.118	−0.111
Personal locus of control	−0.335^***^	−0.333^***^	−0.332^***^	−0.333^***^
Demographic covariates				
Female	0.050	0.038	0.046	0.037
Non-white	−0.129^*^	−0.126^*^	−0.124^*^	−0.124^*^
Age	0.004	−0.006	0.007	−0.004
Coupled	0.018	0.018	−0.010	0.008
Parent	−0.005	−0.016	−0.071	−0.039
High educational debt	0.173^**^	0.134^*^	0.175^**^	0.139^*^
High household income	−0.101	−0.097	−0.097	−0.096
STEM experience covariates				
Postdoc year (Year one)	−0.099	−0.064	−0.097	−0.066
Science community	−0.063	−0.066	−0.058	−0.064
Science efficacy	−0.059	−0.071	−0.046	−0.065
Has a faculty mentor	−0.055	−0.031	−0.056	−0.033
Experienced success	0.048	0.043	0.050	0.045
Experienced unfair treatment	0.142^*^	0.090	0.119	0.087
Adjusted R-Square	0.39	0.44	0.41	0.44
Change In R-Square		0.05^***^	0.02^*^	0.05^***^

N=215. * *p<0.05;*

** *p<0.01;*

*** *p<0.001.*

The standardized betas suggest the most powerful explanatory variables are two of the positive health holdings, general health and internal locus of control. These two health covariates are predictive, negatively, of poor mental health conditions. The healthier the person is generally, the less likely they are to suffer from anxiety (*B*=−1.91, *β*=−0.24, *p*<0.001), and the more the person reports an internal locus of control, the less likely they are to suffer from anxiety (*B*=−0.44, *β*=−0.34, *p*<0.001).

Surprisingly, but affirming the bivariate analysis, few of the usual demographic covariates (gender, age, and income) are significantly predictive of generalized anxiety symptoms. Race does seem to matter, as we see that non-Whites in this population are less likely to suffer from anxiety than Whites (*B*=−1.31, *β*=−0.13, *p*=0.03). This aligns with other research on what has come to be called a minority mental health paradox, where racial-ethnic minorities report better mental health than non-Hispanic whites despite experiencing conditions that might seem less conducive to psychological well-being (Williams and Earl, 2007). The other demographic variable associated with poor mental health is high educational debt. When respondents have debt over $40,000, they are more likely to report symptoms of anxiety (*B*=2.18, *β*=0.17, *p*<0.001).

Unlike the bivariate findings, among the STEM experience covariates, only experiences of unfair treatment are associated with mental health outcomes. If the respondent has experienced unfair treatment, they are more likely to have symptoms of anxiety (*B*=0.16, *β*=0.14, *p*=0.01).

#### Work-Life Conflict

In Table 2, Column II, we show that work-life conflict is predictive of anxiety, even with the various controls represented in the reduced model; hypothesis 2a is supported. The higher the respondents’ work-life conflict, the more likely they are to experience anxiety (*R*^2^=0.438, *B*=0.38, *β*=0.24, *p*<0.001). Adding work-life conflict increased the explanatory power of this model by 5 %. General good health (*β*=−0.20, *p*<0.001), personal locus of control (*β*=−0.33, p<0.001), being non-White (*β*=−0.13, *p*=0.02), and high educational debt (*β*=0.13, *p*=0.01) remain statistically significant in the full anxiety model; unfair treatment no longer does.

#### Life-Work Conflict

In Table 2, Column III, we reveal that life-work conflict is also predictive of anxiety. The higher the respondents’ life-to-work conflict, the more likely they are to experience higher levels of anxiety (*R*^2^=0.408, *B*=0.25, *β*=0.16, *p*=0.02); hypothesis 2b is supported. Adding work-life conflict increased the explanatory power of this model less than adding work-life conflict (*F*=0.02). General good health (*β*=−0.20, *p*=0.00), personal locus of control (*β*=−0.32, *p*<0.001), being non-White (*β*=−0.12, *p*=0.02), and high educational debt (*β*=0.18, *p*<0.001) remain statistically significant in the full anxiety model; again unfair treatment no longer does.

When work-life conflict is added to the life-work conflict model (Table 2, Column IV), life-work conflict is no longer significant; it is no longer predictive of anxiety. This suggests that the real driver of the anxiety postdocs feel regarding imbalances between their work responsibilities/time and their home responsibilities/time is the difficulties imposed on their home lives by their work. That is not to say life-work conflicts are irrelevant; they do reduce the explanatory power of the work-life conflicts slightly. But given that more postdocs experience work-life conflict (62%) than experience life-work conflict (26%), it makes sense that more of that third of postdocs who suffer anxiety symptoms likely do so because their work impinges on their home/life.

## Discussion

STEM postdoctoral trainees are situated in an unusual space in the academic pipeline. They are neither full-time students like they were when pursuing the doctorate and they are not full-time workers as they might be if they had taken a faculty or industry position upon graduating. Instead, they are more like medical residents and house staff, still in a training position where they work full-time employee-like hours either learning new skills, deepening their knowledge in material/skills gained in graduate school, or accumulating additional credentials (e.g., publications) necessary for a competitive application for employment beyond the postdoc appointment. Like many full-time workers, it is likely that these kinds of working trainees find it difficult to avoid conflicts between their vocational responsibilities and their leisure, family, and avocational pursuits. We maintained that the majority of postdocs experience incursions of their work on their lives and, presumably, their lives on their work. Like other research on these conflicts, we expected postdocs who struggle with these related phenomena to also incur some mental health challenges as a result.

Our findings show that postdocs do experience conflicts between their work responsibilities and their non-work responsibilities, but the two are not simply opposites of each other. The majority of postdocs report that the demands of their job interfere with their home and family life, but other than parents, the majority of them do not seem to experience incursions of their home/family life on their work responsibilities. While we expected singles to report fewer incursions, only a quarter of coupled postdocs report life-to-work conflicts. The bigger problem, for those seeking a balance between life and work, is children: Nearly 60% of postdoc parents report life-to-work conflicts with most of those suggesting the conflicts are quite significant relative to what others experience.

Unless one has children, it appears that romantic partners (“partners”) do not make serious demands on one’s time such that postdocs feel that those demands interfere with their ability to meet the responsibilities of their job. This makes sense as partners are likely engaged in their own work responsibilities (79% of our respondent’s partners have a full-time job) that overlap with the work hours of the postdoc. Those postdocs (18% of our sample) with children experience less structure and control over their non-work time, and it likely encroaches on the time supposedly dedicated to their work responsibilities. If, as our questions ask, childcare affects one’s ability to focus only on work when at work, ability to get to work on time, or (as is common in STEM postdoc appointments) working beyond the regular 9 to 5 schedule, those postdocs are going to suffer more incursions of their life’s responsibilities on their work responsibilities.

As research on other workers suggests, these conflicts have consequences; workers with work-life and life-work conflicts have increased mental health difficulties. We show that the higher one’s work-to-life conflicts and the higher their life-to-work conflicts, the higher the degree of anxiety they report. These relationships persist even when we control for general health and protective attributes like dispositional optimism. Like prior research on other workers (Frone, 2000), we do not show that gender moderates this relationship; women postdocs do not differ from men in the amount of work-life/life-work conflict they experience and do not differ in the amount of anxiety they experience as a result of it.

## Limitations

This study is not without its limitations. We recognize that the generalizability of this study is limited by the fact that our conclusions are drawn from a non-random sample of the entire postdoc population (including immigrant postdocs). While the sample we used is nearly representative (once gender weights are applied) of the domestic postdoc population, we cannot be certain of ways foreign-national postdocs – who make up nearly 60% of US STEM postdocs – experience work-life or life-work conflict and the impact of those conflict on their mental health. That said, we do not have any reason to believe that the kind of work they are engaged in is any different in these STEM departments than the work domestic (i.e., US citizens and permanent residents) are doing. The work-to-life dynamics may be the same, but the life-to-work dynamics might differ. Certainly, our understanding of these phenomena would benefit from applying our analysis to this larger STEM postdoc population.

Another limitation is our inability to make any claims about causality, that is, whether having work-life or life-work conflict leads to, rather than is simply predictive of, poor mental health. The current study is a cross-sectional analysis and the ordered relationships between the experiences of these imbalances and mental health cannot be established. A longitudinal study would be more appropriate for establishing a causal relationship. Nevertheless, we contend that the positive relationship between the two suggests experiences of one would, at least, be accompanied by the experiences of the other.

## Conclusion and Recommendations

These limitations aside, the implications of our findings are especially important for guiding institutions on the structure of the postdoc appointment. These findings support the arguments of prior research that work-life imbalance decreases well-being and is therefore a likely contributor to high burnout and low retention in the academic pipeline. In fact, further analysis of our data reveals that postdocs who experience high levels of work-life conflict are less interested in pursuing careers as either research/teaching-intensive or teaching-intensive-only faculty. Even though 90% say that careers in the academy have more autonomy and flexibility than non-academic careers, 87% also believe that non-academic careers offer better work-life balance. Few STEM postdocs have any experience with non-academic employment – only 28% do and half of those were not working in STEM environments – so it is clear that they are basing these impressions almost entirely on their current experience in their postdocs and, likely, observing the faculty they work with and around.

While there appear to be no statistically significant differences between White and Non-White STEM postdocs or between men and women in the degree to which they experience either work-life or life-work conflict, we argue that the broad patterns this project reveals join other elements (e.g., discrimination) in reducing the appeal of an academic career in STEM to underrepresented minorities and women. This and other disamenities make it more difficult for us to broaden participation in STEM careers and diversify the professoriate.

There are always two approaches to issues like these: One involves changing structures and the other involves advising trainees/workers on how to manage difficulties while waiting for structures to change. Our review of the work-life conflict literature and our conversations with the postdocs who provided the survey data this report is based on led us to the following recommendations for those responsible for structural changes (e.g., postdoc office administrators, principal investigators and mentors) and for the individual postdocs themselves. While there are important structural changes that might benefit particular kinds of postdocs (e.g., more childcare for postdoc parents), most postdocs would not be affected by those changes. Therefore, our suggestions will speak to more global issues. In order to give readers more insight into how postdocs describe their experiences, we provide quotes from some of our respondents to provide some context for the suggestions.

### Structural Recommendations

“I actually had to detach myself from that PI because I was, like, ‘you're pressuring me too much on working these 12h days.’ I’m getting sick. I don't want this. This is not for me.”

“This was the kind of lab where you were just expected to produce, and if you weren't producing, you were going to get in trouble. Any time the PI had a whim, you were going to do it and there was no regard for your interests or burnout or anything, you know?”

“There was not even one weekend that I stayed home and didn't come to work, not even one. I was working on President’s Day, all the vacation days I was here in order to be able to manage to do all these things.”

The research on work-life conflict is clear: In order for workers to balance their work responsibilities and their non-work responsibilities/experiences, they must have the support of supervisors who understand how important it is to balance these things and who take steps to help them achieve that balance (Jansen et al., 2003).

Academic research is entrepreneurial (Casati and Genet, 2014; Price et al., 2018; Pitt et al., 2020) and as such can take on the same problematic attributes – problematic for both employer (PI) and employee (postdoc) – that we see among new commercial entrepreneurs: having to take on (or delegate) multiple roles/responsibilities, negotiating often ambiguous performance and productivity expectations, and “always being on the job.”^10^ The autonomy, flexibility, and uncertainty that characterizes academic science (Bailyn, 2003; Fox et al., 2011) often has a knock-on effect, for postdoctoral supervisors themselves, of poor work-life balance and permeability between their work and their non-work lives. They then model poor work-life balance and, worse, impose similar expectations for limitless labor onto their postdoctoral trainees.

Our respondents point to three primary causes for their difficulty constraining work time so it does not take over their lives: the constant and ever-evolving demands on their time at work, the amount of time they believe is required to meet those demands “successfully,” and (ironically) the flexibility in scheduling work that is common in academic spaces. All three of these catalysts for work-life conflict can be managed by structural changes either imposed by institutions or adopted by postdoctoral supervisors (PI’s) and advisors.

Some changes can be adopted simply by recognizing that, regardless of their formal categorization as employees (often “postdoctoral associates”) or non-employees (often “postdoctoral fellows”), most postdocs are *trainees.* They are not only engaged in collaborative research with the postdoctoral supervisors, but they are also supposed to be using these positions as launching pads to independent research careers. Like graduate trainees, they are compelled to engage in multiple tasks with vague metrics for measuring success/completion. Trying to meet both their and their supervisors’ expectations leads to long hours and heightened anxiety about whether or not enough hours are being spent. Departments and faculty supervisors must recognize that these sometimes competing performance pressures cause postdoc trainees to lose sight of the boundaries between their work hours and their non-work hours. They must resolve to help postdocs constrain their work to some reasonable number of hours akin to what faculty themselves work or, we argue, better than that.^11^

The fact that work-life conflicts seem to have mental health ramifications means that institutions must provide support, including counseling and psychological services, for postdocs just as they do undergraduate and graduate trainees.

### Individual Recommendations

“This is a job where day after day, you fail, fail, fail. It’s important for me to just leave work at work. If the experiments fail, they fail, but then I go home and still enjoy my life outside of the workplace and not feel the pressure of, ‘Well, you are failing, so get back to work.’”

“Science is a weird job where you may not always be putting in a ton of work hours, but you put in a huge amount of thought hours. I was working 10h days, but I was also thinking about stuff at home. A huge amount of thought hours made it feel like I was working all the time.”

“I feel like what happens with people in postdocs is they're just working and working and working and working. They’re thinking about that vacation they're going to take, but they don't take it. They love to paint, but haven’t painted in three months. I think it’s really important to try, even if it’s not as much as you’d like, to fit in some of those things that bring you joy.”

We have two recommendations for postdocs themselves: engage in an exercise of personal role redefinition and create boundaries around your work and your life.

Just as it critical for PIs to recognize that their postdocs are trainees and not employees, it is also important for postdocs to see themselves in the same light, but with a twist. Because most postdocs are structured in employee-like ways, with responsibilities to produce some “product” either for themselves or for/with their PIs, there is room for reframing scientific work as requiring unending effort (i.e., work hours) and rumination (i.e., thought hours). Creating concrete tasks with narrow measures of success, especially if done in consultation with one’s PI, enables postdocs to end each work period feeling like they did “enough” rather than constantly feeling like there’s more to be done and it has to be done now. As postdocs put great emphasis on how their PIs experience work-life balance, future research on the impact of postdocs’ observations of their PIs’ work arrangements and the PIs’ perceptions and experiences of their work may further explain the relationship between work-life conflict and mental health of postdocs.

Ironically, the fact that postdocs *are* trainees, makes it hard for them to transition from practices they engaged in as undergraduate and graduate students, practices that do not work as well in the liminal space they are in. The uncertainty students feel about their performance, often caused by delayed positive evaluations of that performance, causes them to think of effort – measured by time spent engaged in something – as the best evidence of commitment and competence. There is pressure to always look like, and for many always be, “actively engaged workers.” Some research shows that this uncertainty even leads people to volunteer to work more hours than prescribed for them as a way to look and feel like a serious worker (Sharone, 2005).

Postdocs who have worked in full-time jobs at some point since receiving their baccalaureate degrees suggest that the break from the student-role, with all of its delays in assessments of “good and completed work,” enabled them to better craft a sense of themselves as workers with a concrete set of responsibilities to a concrete set of stakeholders limited to a concrete space and amount of time in which to complete them. Gaining some semblance of control of work-life balance in those spaces carried over into their graduate and postdoctoral traineeships. They could see the potential for encroachment more clearly and, when empowered to do so by supervisors who would listen, could head it off by monitoring and circumscribing that encroachment.

Again, the autonomy and flexibility that characterizes academic work often comes with a cost. Without the constraints of hourly wages, offices that shut down at 5pm and concrete evaluations of productivity (i.e., you have made X number of things this week), it can become very easy to ignore the way work expands to fill the vacuum of a boundaryless non-work life. Families – even children – can be good for balance. They often force postdocs to create boundaries around work. But even then, postdocs tell us that they take work home and continue working on it after their children are asleep. Single and child-free postdocs have to be even more vigilant without family “allies” in their attempts to reduce work’s encroachment on their non-work lives and leisure.

It is important that postdocs learn segmentation practices where they create, and maintain, hard boundaries between work and their “life.” Attempts to reduce the amount of contact one has with work when engaged in life can go a long way in reducing both the experience of role-conflicts and the sense that the boundaries between the roles are blurry. Boundary marking, or making decisions about ways work can go “this far and no farther,” becomes especially important given ways communication technology (e.g., email and smart phones) enables work to follow us into our lives if not constrained.

One important boundary marker can be differentiating between the spaces where work and life happens. For most science trainees, the bulk of their work takes place in an on-campus laboratory or in on-campus offices. Deciding that they will only work in spaces dedicated to their work as postdocs and not allow themselves to engage in work-related activities (or accept work-related contacts) in their home is an important step in creating a boundary between work and life. Often academic workers, postdocs and faculty mentors alike, create dedicated office spaces in their homes. We believe this is a mistake. Just as most employers are still resistant to create spaces for “life” (e.g., daycares, tv lounges, and exercise rooms) in work contexts, postdocs must endeavor to resist creating room (and, literally, rooms) for work in their living spaces.

Notably, the COVID-19 pandemic changed our usual work patterns (Ashencaen-Crabtree et al., 2021; Matulevicius et al., 2021; Möhring et al., 2021) suddenly forcing many science trainees to shift all of their work into their homes. While it remains uncertain how much the new patterns of making work arrangements more flexible (and therefore, less predictable) and shifting more work into workers’ homes will persist, it is important to recognize that even prior to the pandemic, both of these patterns were clearly causing problems with work-life balance and boundary marking (Hayman, 2009; Felstead and Henseke, 2017). Our hope (and our recommendation) is that faculty supervisors/employers do not casually embrace these patterns as norms without counting the costs to their trainees’ health.

Creating boundaries between work and life/leisure cannot be left to chance. As the quotes suggest, the culture of academic science seems to expect and privilege over-work. These cultural norms – this is not necessarily a function of the “nature” of science – can be managed and, if we all consider the health consequences of them, undone. Just as scholars (e.g., Cech and Blair-Loy, 2014; McGee, 2021) are pushing us to consider ways the culture of academic science are pushing people of color and women away from academic careers, we hope this project further prompts administrators, faculty, and science-discipline associations to think of how this cultural norm – that a minimal non-work life is expected – might be pushing them away as well.

## Data Availability Statement

The raw data supporting the conclusions of this article will be made available by the authors, without undue reservation.

## Ethics Statement

The studies involving human participants were reviewed and approved by Behavioral and Social Sciences IRB Office, Vanderbilt University. The patients/participants provided their written informed consent to participate in this study.

## Author Contributions

RP supervised the project and RP, YT, and IS wrote and revised the manuscript. All authors contributed to the article and approved the submitted version.

## Funding

This research is made possible due to the generous support of the National Science Foundation, Grant no. #HRD-1647196. The findings presented are those of the researchers and do not necessarily reflect the views of the NSF.

## Conflict of Interest

The authors declare that the research was conducted in the absence of any commercial or financial relationships that could be construed as a potential conflict of interest.

## Publisher’s Note

All claims expressed in this article are solely those of the authors and do not necessarily represent those of their affiliated organizations, or those of the publisher, the editors and the reviewers. Any product that may be evaluated in this article, or claim that may be made by its manufacturer, is not guaranteed or endorsed by the publisher.

## Footnotes

^1^Research in this area often refers to these conflicts as conflicts between work and “family” even though many of them, necessarily, analyze the impact of “family” conflicts with work responsibilities experienced by unmarried or uncoupled workers without children. We use a broader term – *work-life* – to capture various ways one’s (home) life might impact or be impacted by their work.

^2^An exception to this is the amount of attention given to work-life conflicts encountered by medical residents, house staff, and surgical fellows (Schwartz et al., 1990; Dorsey et al., 2003; Tambyraja et al., 2008; Glynn and Kerin, 2010).

^3^In all cases, the offices were not allowed to give us names and other details of their postdoctoral population. As a result, we could not constrain the list of invitees to only those postdocs who met our study parameters.

^4^As this project is part of a larger study aimed at broadening participation in STEM training and diversifying the STEM professoriate, we follow the lead of the National-Academy-of-Sciences (2011) in focusing our attention on the physical sciences, the life sciences, engineering, and mathematics only. We, therefore, exclude the social sciences (e.g., sociology, psychology, and history) and social science focused agriculture/conservation postdocs.

^5^The focus of the larger study (i.e., on diversifying the STEM professoriate) led us to exclude foreign postdocs, whose career motivations, pathways to postdocs, and ability to “diversify the professoriate” have been shown to be significantly different from non-foreign STEM doctorate recipients, particularly non-Whites (Amuedo-Dorantes and Furtado, Stephan and Ma, 2005, Zeithammer and Kellogg, 2013).

^6^Respondents who indicated that they were either “married” or “in a committed relationship” were coded as “coupled.”

^7^The mean for this scale is slightly less than 2 which represents “less than once a year”; about 30% of our respondents say they experience unfair treatment more than this. Neither gender nor race is correlated with this variable in our sample.

^8^The correlation between science identity centrality and anxiety is insignificant.

^9^The name refers to Black folk-hero John Henry who ultimately died from a heart attack in an effort to beat a steam-powered rock drilling machine in a race to build a tunnel.

^10^According to a 2016 Bank of the West Small Business Growth Survey, 43% of small business owners say “always being on the job” is one of the biggest challenges they face. Other studies show that 33% of small business owners work more than 50 h a week; 25% work more than 60.

^11^While we see parallels between postdocs and medical residents/house staff, the medical internship regime is *not* a model here. While medical residents are constrained to working a maximum of 80 h a week averaged over a month’s time—which is terrible in itself—the boundaryless of their work (direct patient care, ancillary care, and paperwork) can cause some residents to work more than 120 h in some weeks.
